# Supplementary material for: “Mother in the making”: Motherhood performativity of childless women in rural Pakistan
Source: PLoS One. 2026 Feb 6;21(2):e0326698. doi: 10.1371/journal.pone.0326698 (PMC12880684; doi:10.1371/journal.pone.0326698)
Supplement: Attachment — Submitted filename: Observations.docx [file pone.0326698.s001.docx]

1. **Summary of the Research and Personal Overall Impression**

The research brings out issues that trouble women failing to have children. Motherhood performativity of childless women is evident in rural Punjab. The research reveals that they are seen as incomplete because of childlessness. When they get children (biologically or by adoption) and love them, they will have performed what is socially required for their gender. This study revealed that they are seen as responsible for childless marriages. Men are excused from impotence.

I commend the use of Butler’s Performativity theory as it amplifies the lived experience of involuntarily childless women. The researchers managed to highlight areas that a women must perform to validate her gender.

What I like about this research is the choice of a rural context for the study of involuntary childlessness. This is where the cultural expectations are undiluted by modernisation. The context reveals what a specific people group believe before their culture is influenced by other cultures.

2. **Discussion of Specific Areas for Improvement**

**The Conceptual Framework**

Instead of describing Butler’s Performativity theory in depth, the researchers were supposed to highlight the main key factors and how they are related. In this case, women’s identity or gender in rural Punjab is validated by their performativity. Bearing children in this study is the main “performance” that women can use to validate their gender.

**Research Methodology**

The research methodology section on page 8 and 9 is discussing the research context.

The researchers didn’t highlight the limitations of their study. For instance, the primary researcher was a mother of two children. What effect did that have on the data analysis? Did the self of the researcher have any impact on the presentation of findings? If not, what measures were put in place? Or how was that achieved?

**Data Analysis**

In data analysis, the researchers need to reduce the number of direct quotations of the participants. They can paraphrase them and give their interpretation of the excerpts. In some places, there is very little data analysis but more participant quotations. Readers will need to hear what the researchers make of the collected data.

How is the cited excerpt speaking to motherhood performativity of Punjabi childless women? How does it address the implications of the exhibited behaviours and further reveal the importance attached to motherhood? How does the gendered expectation of mothering a child regulate the lives of these childless women as they work towards becoming mothers?

**Flow of Thought**

The clarity of some of the research findings is clouded by language articulation. While there are some minor typographical errors, there are some portions in the manuscript that will need expert language editing. This will enhance the readability of the article.

The researchers need to allow an interaction of the authors they cite. They can employ using joining words to make the transition from one author to the other smoother. On page 5 and 6, the authors have liberally used direct quotations. They can paraphrase the quotes to avoid monotony.

A minor observation is on use of respondents and participants interchangeably throughout the paper. This article has participants rather than respondents. The target population provided qualitative data.

The section which discusses the bride in limbo (page 15 onwards) is not very clear. The connection between the subtheme (mentioned in the title) and the body needs to be strengthened. The researchers have a good intention; however, more clarity is needed to openly bring out the finding on motherhood performativity in Punjabi childless women.

**Confidentiality**

In the data collection paragraph (page 11, last line), I am concerned about confidentiality issues. If “All transcriptions were rechecked by two language experts,” what did the researchers put in place to protect the participant’s identity? Clarity on that may help

In data analysis, when coding the participants, mentioning their age may reveal their identity to those who may have seen the participants. I suggest a revised coding system.

**Layout of Manuscript**

Page numbers are missing. This is a minor issue that can be rectified.

**References and Intext Citations**

On page 27, reference number 7, the researchers must follow the guidelines for published media (print or online newspapers and magazine articles).

The numbering of references did not follow the order they appear in the text. Authors should refer to the author guidelines for this.

I have a minor observation on intext citations throughout the paper. According to the guidelines, in the text, the reference number must be cited in square brackets. The researchers used parentheses.

The authors must align all other references to the Vancouver reference style.

**Recommendation**

I recommend the article for publication. It presents a crucial matter that needs to be discussed. However, the above revisions can be taken into consideration to strengthen the article. More attention should be given to data analysis. In some places (especially the first section on “The constitution of marriage pointing towards motherhood”, over-quoting the participants repressed in-depth analysis.

3. **Other Points**

Thank you for the opportunity to peer-review an article that addresses a subject that is dear to me. I am willing to look at the revised article.
