# Supplementary material for: “Mother in the making”: Motherhood performativity of childless women in rural Pakistan
Source: PLoS One. 2026 Feb 6;21(2):e0326698. doi: 10.1371/journal.pone.0326698 (PMC12880684; doi:10.1371/journal.pone.0326698)
Supplement: Attachment — Submitted filename: plosone-reviewer.doc [file pone.0326698.s003.doc]

This study focuses on childless women in rural areas of Pakistan and applies Judith Butler's Performatity Theory to explore how they construct a "expectant mother" identity through a series of social behaviors, such as seeking medical treatment, displaying maternal traits, and participating in the care of others' children, under the pressure of a strong reproductive culture, in order to cope with the stigma of being childless.

1.Butler's performative theory has only been briefly summarized, without fully elaborating on its specific connection with maternal construction. Suggest supplementing the connection between theoretical framework and empirical data

2.The fertility rate data of Pakistan is quoted up to 2001 (NIPS) and 2017-18 (PDHS). It is recommended to supplement the data from the past 5 years to reflect the forefront of research

3.The interview outline content or FGD guidance questions are not clearly defined. It is recommended to supplement the appendix or example questions to ensure method transparency.

4.The conclusion mentions that 'women perform motherhood through therapeutic behavior', but does not delve into the power structure behind the behavior. It is suggested to deepen the discussion by combining theory.

5.Only mentioning adoption as an alternative, without analyzing how it challenges/maintains traditional maternal norms.

6.Some terms are used in confusion, it is recommended to unify the terminology and add key concept definitions

7.The conclusion does not propose policy recommendations for rural medical resources or anti stigmatization. It should be explicitly acknowledged that the sample is limited to a single village and the impact of socioeconomic status on treatment choices has not been explored.
