# Supplementary material for: “Mother in the making”: Motherhood performativity of childless women in rural Pakistan
Source: PLoS One. 2026 Feb 6;21(2):e0326698. doi: 10.1371/journal.pone.0326698 (PMC12880684; doi:10.1371/journal.pone.0326698)
Supplement: Attachment — Submitted filename: Response_to_reviewers_auresp_2.docx [file pone.0326698.s004.docx]

**Response to Reviewers**

We are thankful to the reviewers of this paper for their interest and insightful suggestions; we appreciate their effort and valuable suggestions. We have taken all the reviewers' comments on board and tried our best to incorporate them. The responses to the comments are given below, one by one (for each comment).

| **Comment** | **Response** |
| --- | --- |
| **Reviewer # 2** | |
| 1.TheoreticalApplications: While the theoretical framework is well-established, there could be more explicit connections between Butler's concept of "subversion" and the adoption practices described in the fifth theme. This would strengthen the analysis of how women exercise agency within constrained cultural contexts. | More detailed analysis has been added in this theme. See page 26 & 27. |
| 2. Comparative Context: The manuscript could benefit from brief comparisons with findings from studies in other cultural contexts to highlight what might be unique about the Pakistani rural context versus what might be more universal experiences of childless women in pronatalist societies. | This suggestion has been incorporated by adding the studies from different regions and also from Pakistan (See theoretical framework and results sections) |
| 3. Policy Implications: Consider adding a brief discussion about the potential implications of these findings for healthcare providers, community workers, or policymakers working with childless women in rural Pakistan or similar contexts. | A brief discussion has been added in the conclusion part. See page 29 & 30 |
| 4. Intersectionality: Although the paper acknowledges differences in religion, education, and marriage duration among participants, a more explicit discussion of how these factors might intersect to shape women's experiences of childlessness would enhance the analysis. | Since this study is part of a broader project, we initially tried to apply the theory of intersectionality. However, we could not do so because the study had several limitations in terms of its geographical scope and the number of participants. Therefore, a full analysis considering all these factors was not possible, but we carried out as much analysis as was feasible.  (We could only find differences on the basis of marriage duration (women in the initial years focused more on medication while with long duration such as 10 years or more they think about adoption) Others intersections possibly not visible due to sample size taken from a village that is small in number. See page) |
| Overall, this is a well-executed qualitative study that makes a significant contribution to understanding the gendered experiences of childless women in rural Pakistan. The manuscript effectively demonstrates how these women navigate cultural expectations through performances that affirm their maternal identity and desire, even in the absence of biological children. The paper provides valuable insights into the complex interplay between cultural discourses, gender norms, and individual agency in shaping women's lives and identities. | Thank you for the positive comment |
| **Reviewer # 3** | |
| 1.Butler's performative theory has only been briefly summarized, without fully elaborating on its specific connection with maternal construction. Suggest supplementing the connection between theoretical framework and empirical data | This point has been incorporated. See theoretical framework and result sections. |
| 2.The fertility rate data of Pakistan is quoted up to 2001 (NIPS) and 2017-18 (PDHS). It is recommended to supplement the data from the past 5 years to reflect the forefront of research | The latest family planning survey in Pakistan is **the one conducted in 2001.** The most recent PDHS survey is **from 2017–18,** and a **PDS 2020** report was published **based on the PDHS 2017–18 data.**  https://www.google.com/search?q=latest+PDHS+survey+pakistan&sca_esv=ef5f6f2b1a614c94&sxsrf=AE3TifNy3XFW7TQPraA5q66cEyIN_-SCGg%3A1753681791500&ei=fw-HaIWmHqyHkdUP7c6tiQs&ved=0ahUKEwjF4ezh7d6OAxWsQ6QEHW1nK7EQ4dUDCBA&uact=5&oq=latest+UNFPA&gs_lp=Egxnd3Mtd2l6LXNlcnAiDGxhdGVzdCBVTkZQQUgAUABYAHAAeACQAQCYAQCgAQCqAQC4AQPIAQCYAgCgAgCYAwCSBwCgBwCyBwC4BwDCBwDIBwA&sclient=gws-wiz-serp |
| 3.The interview outline content or FGD guidance questions are not clearly defined. It is recommended to supplement the appendix or example questions to ensure method transparency. | The interview guide and FGD guide are attached. See appendix |
| 4.The conclusion mentions that 'women perform motherhood through therapeutic behavior', but does not delve into the power structure behind the behavior. It is suggested to deepen the discussion by combining theory. | Conclusion has been rewritten. See page 27 |
| 5.Only mentioning adoption as an alternative, without analyzing how it challenges/maintains traditional maternal norms. | This theme has been enhanced. See page 25 & 26 |
| 6.Some terms are used in confusion, it is recommended to unify the terminology and add key concept definitions | We added the definition for (a few) terms in the manuscript. |
| 7.The conclusion does not propose policy recommendations for rural medical resources or anti stigmatization. It should be explicitly acknowledged that the sample is limited to a single village and the impact of socioeconomic status on treatment choices has not been explored. | This comment has been incorporated. See conclusion page 29 |
| **Reviewer # 4** | |
| This research is novel and innovative. The methodology is sound and well-described. However, there are some issues which need addressing in order to improve the quality of the manuscript. Please see my recommendations below. | Thank you |
| 1. The manuscript would benefit from one more professional edit. There are some small expression issues that need to be ironed out. | The manuscript has been entirely rechecked by a professional editor. |
| 2. There are several points in the manuscript where the research is referred to as ‘my study’ even though there is more than one author listed.  Also, in the analysis section, it is revealed that the first author conducted the analysis by themselves. Please correct me if I am wrong about this. It is unclear to me what the contribution of the other authors consists of. It should be more evident in the manuscript how all contributed. | This comment has been incorporated.  The contributions of all authors has been mentioned. See page 14 |
| 3. The abstract highlights the main contribution of the article as showing how ‘societal discourses shape women’s lives.’ However, this is not necessarily new in the literature. What is the study adding to what we know about motherhood, performing gender, involuntary childlessness? This should be better emphasised in the abstract, introduction, literature review and conclusion. | The suggestion has been incorporated in all parts of the manuscript. |
| 4. Related to the point above, there is too much dwelling on performativity and explaining Butler in the literature review. There should be more connections made with the broad literature on gender performativity, motherhood expectations, and infertility. What do other studies say about these topics and how can this research contribute to these conversations even if they are not necessarily located in Pakistan. | The theoretical framework and conclusion sections have been enhanced. |
| 5. I am wondering about the rationale behind broadening the childless category. Is it because all participants experienced the pressures of motherhood at some point? This should be specified early in the manuscript. | There are no different types of childlessness. Actually, it refers to various life stages of women who could not bear a child. It is true that women are under pressure to achieve motherhood right after marriage, and it increases with the passage of time (if a woman could not achieve motherhood). |
| Below table 1 there are two paragraphs that are an exact copy of each other. | One paragraph has been removed. |
| 7. Theme titles are long and convoluted. I recommend shortening them to make the key message of each theme clearer. | The theme titles have been shortened as much as possible |
| 8.The conclusion needs to be strengthened. It does not discuss any of the broader implications of the findings. This is a shame because the analysis is excellent. I already recommended that more relevant literature should be added to the review in the first part of the manuscript. The conclusion should connect to this literature (on motherhood, childlessness, etc.) by telling us how the findings contribute to scholarly work that is related, but not specific to Pakistan. There is an opportunity to strengthen the relevance of your findings, and this should not be missed. | The conclusion has been enhanced and linked to both childlessness and motherhood. |

**Appendix**

**Interview guide**

This interview guide is showing the questions that are relevant to this study.

1. How long have you been married?
2. After how long into your marriage did people start asking about having a child?
3. Who was more interested in your pregnancy?

Family, neighbours, friends, community

4. Which side of the family was more interested in your pregnancy—your parents or your in-laws? Why?
5. What type of questions did they (community members) ask you?
6. How did you feel when they asked questions about your possible pregnancy?
7. How did you handle the situation?
8. Did you receive any support to handle the situation?
9. When did they (community members) start asking you questions directly?
10. With whom did you share your feelings??
11. What did you dislike most?
12. By when you feel that you should have a child?
13. What did you do at that time?
14. What type of efforts did you make?
15. Have you used any type of treatment?
16. How long did you use the treatment?
17. Whom did you consult first?
18. Who told you about that healer/treatment?
19. Did anybody support you in consulting the healer?
20. How costly was the treatment?
21. Did you try a 2^nd^/3^rd^/4^th^ treatment?
22. For how long have you been using the treatment?
23. Which types of treatments have you used?
24. Did you ever think of stopping the treatments?
25. Do people ask your husband questions about this?
26. How do you express your feelings about these years of treatment?
27. How did your feelings change initially, after one year, and so on?
28. How did your feelings or expressions change from the beginning to the present?
29. How are you handling the situation at present?
30. How do you remember all these treatments one by one?
31. Did you ever feel satisfied at times?
32. How did people’s behaviour change towards you and your life over time?
33. What did you feel and notice during discussions?
34. Can you describe how you felt at the time of your marriage?
35. When did you first hear comments about having a child?
36. What were your friends and family saying?
37. What were your in-laws saying?
38. How did you feel when you had to talk about the treatments and your efforts repeatedly?
39. Why did you do that repeatedly?
40. How do/did you feel about other people’s children?
41. Do you love other people’s children?
42. How do/did your nephews and nieces behave towards you?
43. Have you ever thought about adoption?
1. **If Yes,**

- What made you consider adoption, and why?? Why
- Can you describe the adoption process??
- How do you feel today?
- How does the community treat you now?
- Have you noticed any changes in your life now??
- In terms of satisfaction, family behaviour, community behaviour,

2. **If No,**

- Why?
- Is there any possibility of adoption in the future, or reasons for not adopting

**Guide Focus Group Discussion**

1. How important is a child for a woman?
2. What is the perception of a woman without a child?
3. Who is more concerned about a woman having a child?
4. When does it become necessary to have a child?
5. **When does the community first remind a woman to become a mother**?
6. How does the community remind a woman about having a child for the first time?
7. Are there specific occasions or rituals involved?
8. Who is the decision-maker regarding when a woman should have a child?
9. Who scrutinises or judges the woman in this situation?
10. How long should the family wait?
11. What should the family do if a woman does not conceive within the given time?
12. How can a family support a woman?
13. What should a woman do to conceive?
14. What types of treatments can she use?
15. How long should a woman continue treatment(s)?
16. What is the appropriate time to have a child after marriage?
17. What is considered a normal versus abnormal time period?
18. What is mamta?
19. How does the community perceive ‘mamta’ in a woman?
20. What are the characteristics of a good mother?
21. What characteristics show a woman’s desire for motherhood?
22. Can a woman (or couple) adopt a child?
23. Who is important in this type of decision-making?
24. **Where can a woman (or couple) adopt a child from**?
25. Can a foster home provide a child to the woman (or couple)?
26. What should a woman (couple) consider while adopting?
